# Comparison of preparation methods of rat kidney single-cell suspensions

**DOI:** 10.1038/s41598-024-53270-2

**Published:** 2024-02-02

**Authors:** Tiantian Wang, Wanjun Shen, Lin Li, Haoran Wang, Min Zhang, Xiangmei Chen

**Affiliations:** Department of Nephrology, First Medical Center of Chinese PLA General Hospital, National Key Laboratory of Kidney Diseases, National Clinical Research Center for Kidney Diseases, Military Logistics Research Key Laboratory of Field Disease Treatment, Beijing Key Laboratory of Kidney Disease Research, Beijing, 100853 China

**Keywords:** Kidney, Cytological techniques

## Abstract

Preparation of kidney tissue single-cell suspensions is the basis of single-cell sequencing, flow cytometry and primary cell culture, but it is difficult to prepare high quality whole kidney single-cell suspensions because of the complex structure of the kidney. We explored a technique called stepwise enzymatic digestion (StE) method for preparing a single-cell suspension of rat whole kidney tissue which contained three main steps. The first step is to cut the kidney into a homogenate. The second step is the digestion of renal tubules using Multi Tissue Dissociation Kit 2 and the last step is the digestion of glomeruli using type IV collagenase. We also compared it with two previous techniques, mechanical grinding method and simple enzymatic digestion method. The StE method had the advantages of high intrinsic glomerular cells and immune cells harvest rate, high singlets rate and high cell viability compared with the other two techniques. In conclusion, the StE method is feasible, highly efficient, and worthy of further research and development.

## Introduction

Preparation of single-cell suspensions is an essential part of scientific research, such as in culturing of primary cells^1^, pretreatment before flow cytometry^2^ and high-throughput sequencing^3^, and the preparation of functional organoids^4^. However, the complexity of the preparation process of single-cell suspensions and the quality of the obtained cells are different for different organs. The amount, viability dispersion and fragmentation of cells in the prepared single-cell suspension will affect the feasibility of the follow-up experiment and the credibility of the experimental results.

The kidneys play important roles in maintaining water, electrolyte and acid‒base balance; clearing endogenous and foreign metabolites; regulating blood pressure; secreting hormones; and maintaining homeostasis of the internal environment^5,6^. Coordinated interactions among different cell types in the kidneys are essential for renal function. The kidneys consist of the renal interstitium and the renal parenchyma, the latter of which includes nephrons and collecting ducts. Nephrons are composed of renal corpuscles (themselves composed of glomeruli and renal vesicles) and renal tubules. The complex structure of the kidney involves various cell types, including epithelial cells, endothelial cells, mesangial cells, mesenchymal cells and their subtypes. It also includes immune cells that circulate and reside in the kidneys^7^. To explore renal function, the role of each cell type in this complex system must be determined. More than 80% of renal cortical cells are tubular epithelial cells, which dominate single-cell maps of the kidneys and cannot be ignored in kidney studies at the single-cell level. However, because of their large numbers, tubular epithelial cells can easily obscure other cell populations^8^. Although the glomeruli make up only 1–1.5% of the kidney volume^9^, they play an important role in filtration. Changes in the number, structure and function of podocytes, mesangial cells and endothelial cells in the glomeruli have always been important topics in research on kidney diseases.

Research on kidney tissue at the single-cell level has gradually become popular. The research thus far has not been limited to independent studies on glomeruli, renal tubules or interstitial cells. Researchers have also sought to obtain single-cell suspensions of all cells in intact kidney tissue to obtain the proportions of the various types of cells and to try to discover new cell types. However, the methods used to prepare single-cell suspensions have been different in each study. In addition, similar studies have led to different conclusions. These differences have probably been caused by incomplete or excessive digestion of kidney tissue during the preparation of the single-cell suspensions, which caused partial cell damage and interfered with the experimental results^10^. To improve the acquisition rate and survival rate of kidney-derived single cells after digestion, a method for preparing single-cell suspensions of whole kidney tissue was developed in this study.

## Methods

### Animals

All experimental methods and protocols were performed in accordance with the Animal Research: Reporting of In Vivo Experiments (ARRIVE) guidelines and were approved by the Chinese PLA General Hospital Animal Care and Use Committee. Wild-type Wistar rats (200 g ± 20 g, male, purchased from Beijing SPF Biotechnology Co., Ltd., China) were adaptively fed using a 12-h day/12-h night cycle for 1 week with freely accessible food and water. The rats were anesthetized and euthanized by intraperitoneal injection of 2% sodium pentobarbital. The study followed the Guide for the Care and Use of Laboratory Animals published by the United States National Institutes of Health (NIH publication, 2011 Revision).

## Equipment and reagents

### Equipment

GentleMACS Octo Dissociator with Heaters (Miltenyi Biotec, 130-096-427, Germany) gentleMACS C Tubes (Miltenyi Biotec, 130-093-237).

TC20 Automated Cell Counter (Bio-Rad, 1450102).

Cell Counting Kit dual-chambered slides (Bio-Rad, 1450003).

NovoCyte (Agilent, CA, USA).

Moflo XDP (Beckman Coulter, CA, USA).

### Reagents

Multi Tissue Dissociation Kit 2 (MTDK2, Miltenyi Biotec, 130-110-203).

Collagenase Type IV (Gibco, 17104-019, NY, USA).

Debris Removal Solution (Miltenyi Biotec, 130-109-398).

Red Blood Cell Lysing Buffer (Sigma-Aldrich, R7757, Germany).

Cell staining buffer (BioLegend, 420201, CA, USA).

Trypan blue (Gibco, 15250061).

Zombie NIR Dye (BioLegend, 77184).

### Single-cell suspension preparation

Rats were anesthetized by intraperitoneal injection of 2% pentobarbital solution at a volume of 2 μL/g. Immediately after the complete kidney tissue was removed, it was placed in precooled saline and weighed. The experiment was divided into three groups, namely, the mechanical grinding method (MG) group, simple enzymatic digestion (SE) group and the stepwise enzymatic digestion (StE) group. The kidney tissues of each group were pretreated. Briefly, 1 g of kidney tissue was cut with a sterile scalpel and placed in a 5 mL EP tube. Then, 200 μL of precooled HBSS^−/−^ was added to the tube, and the tissue was sufficiently clipped for homogenization with ophthalmic scissors. All operations were performed on ice.

### Mechanical grinding (MG) method

A 10 cm sterile petri dish was prepared and placed on ice, and the tissue homogenate was placed in a 70 μm cell strainer. The tissue homogenate in the cell strainer was ground into the petri dish with a grinder, and the tissue was repeatedly washed with precooled PBS solution and ground until all the tissue had been filtered. The filter liquid was collected. Another 10 cm sterile petri dish was prepared and placed on ice. The filter liquid was passed through a 40 μm cell strainer, the remaining tissue filter liquid was ground into the petri dish with a grinder, and the filter liquid was collected.

### Simple enzymatic digestion (SE) method

Based on the methodology of previous studies and according to the instructions^6^, an MTDK2 was used. For each 1 g of tissue, 5 mL of MTDK2 reagent preheated to 37 °C was added. The tissue homogenate and MTDK2 reagent were thoroughly mixed, placed in a gentleMACS C tube. The tube was then placed into a gentleMACS Octo Dissociator with Heaters. The 37C_Multi_E program was run (stirring and digestion at 37 °C for 60 min). The digested tissue suspension was collected and filtered through a 40 μm cell strainer, and an equal volume of 10% FBS prepared with PBS was added to terminate digestion. The mixture was centrifuged at 300×*g* and 4 °C for 5 min. The supernatant was removed, and cell staining buffer was added to resuspend the cells.

### Stepwise enzymatic digestion (StE) method

This method involved two-step enzymolysis. The first step used an MTDK2; to each 1 g of tissue, 5 mL of MTDK2 reagent preheated to 37 °C was added. In the second step, type IV collagenase was added. MTDK2 reagent was prepared according to the manufacturer’s instructions, and collagenase IV stock solution was prepared at a concentration of 10 U/μL. Firstly, the tissue homogenate and MTDK2 reagent were thoroughly mixed and placed in a gentleMACS C tube. The tube was then placed into a gentleMACS Octo Dissociator with Heaters and stirred and digested at 37 °C for 30 min. After this procedure, the C tube was removed, and the type IV collagenase stock solution was added to the tissue digestive fluid at 1:50 to a final type IV collagenase concentration of 200 U/mL. Then, the Dissociator was run at 37 °C for 30 min again. The digested tissue suspension was collected through a 40 μm cell strainer, and an equal volume of 10% FBS prepared with PBS was added to terminate digestion. The mixture was centrifuged at 300×*g* and 4 °C for 5 min. The supernatant was removed, and cell staining buffer was added to resuspend the cells.

All three methods are followed by debris removal and red blood cell lysis according to the specifications.

### Cell morphology observation

After gentle aspiration and mixing, 50 μL of cell suspension from each group was drawn up using a sterile pipette tip and dropped onto a petri dish. The suspensions were observed and photographed under an inverted microscope.

### Calculation of cell number and viability

10 μL cell suspension was mixed with an equal volume of trypan blue and dropped on cell counting slides. Then the cells were counted on a TC20 Automated Cell Counter.

### Flow cytometry

After counting, 1 × 10^6^ cells were extracted from the single-cell suspension of each group, and 100 μL of cell staining buffer was added. Zombie NIR Dye was used for dead or live cell staining. Briefly, 0.2 μL of the dye was added to each tube, and the mixture was incubated for 10 min away from light at room temperature. Afterward, 2 mL of PBS was added to wash off the dye 2 times with centrifugation at 500×*g* for 5 min. Then, the supernatant was removed, and 100 μL of cell staining buffer was added to resuspend the cells. Primary antibodies or secondary antibodies were added, and the cells were stained at room temperature for 20 min. Then, 2 mL of PBS was added to wash off the antibodies 2 times with centrifugation at 500×*g* for 5 min. Then, the supernatant was removed, and 500 μL of PBS was added to resuspend the cells. The samples were run on NovoCyte and analyzed using FlowJo 10 software. The cells were sorted in aseptic environment and run on Moflo XDP. The centrifuge tubes were blocked using PBS containing 10% fetal bovine serum at 4 ℃ for 12 h before cell collection and then added with 2 mL RPMI-1640 medium to collect cells. Three samples of each cell type were collected and 2 × 10^6^ cells were collected as one sample which is used for qRT-PCR. The antibodies were as follows: FITC anti-rat CD45 (BioLegend, 202205), anti-PDGFR beta (Proteintech, 13449-1-AP, IL, USA), CoraLite647-conjugated goat anti-rabbit (Proteintech, SA00014-9), PE-conjugated anti-CD10 (Bioss, bs-0527R-PE, China), anti-Nephrin (Abcam, ab216692, UK) and anti-CD31 (Proteintech, 28083-1-AP).

### qRT-PCR

We used TRIZol (Invitrogen, USA) to extract RNA from samples according to the manufacturer’s instructions. The RNA was then reverse transcribed into cDNA using ProtoScript II First Strand cDNA Synthesis Kit (New England Biolabs, USA), and qRT-PCR was performed using SYBR Select Master Mix (Applied Biosystems, USA) according to the manufacturer’s instructions. The primers used are listed in the Table 1.

**Table 1:** The sequences of forward and reverse primers used for qRT-PCR.

Gene	Orientation	Sequence (5′-3′)
*18 s*	Forward	GTAACCCGTTGAACCCCATT
	Reverse	CCATCCAACGGTAGTAGCG
*Mme*	Forward	AGCATCATGGTCTTGGTCTGT
	Reverse	CCCAAATCCTGTCACACCGA
*Pecam1*	Forward	CAGCCATTACGACTCCCAGA
	Reverse	GAGCCTTCCGTTCTCTTGGT
*Pdgfrb*	Forward	ACAGTCCCGGCTACCCTATC
	Reverse	GCCCCTCCTCACTCCAAAAG
*Nphs1*	Forward	ACTGGTTCGTCTTGTCGTCC
	Reverse	ACCCCGTTTTTGGTCCAAGT
*Ptprc*	Forward	TCGGCCCAGAAGTCTTTGTC
	Reverse	GAGATGCTTGGGGGTGTGAA

### Statistical analysis

Data from more than three independent experiments were represented as scatter plots with bars and analyzed by unpaired t-test or one-way ANOVA using SPSS Statistics 23 software. Differences were considered statistically significant at *p* < 0.05.

## Results

### Morphological observation

**Figure 1:**
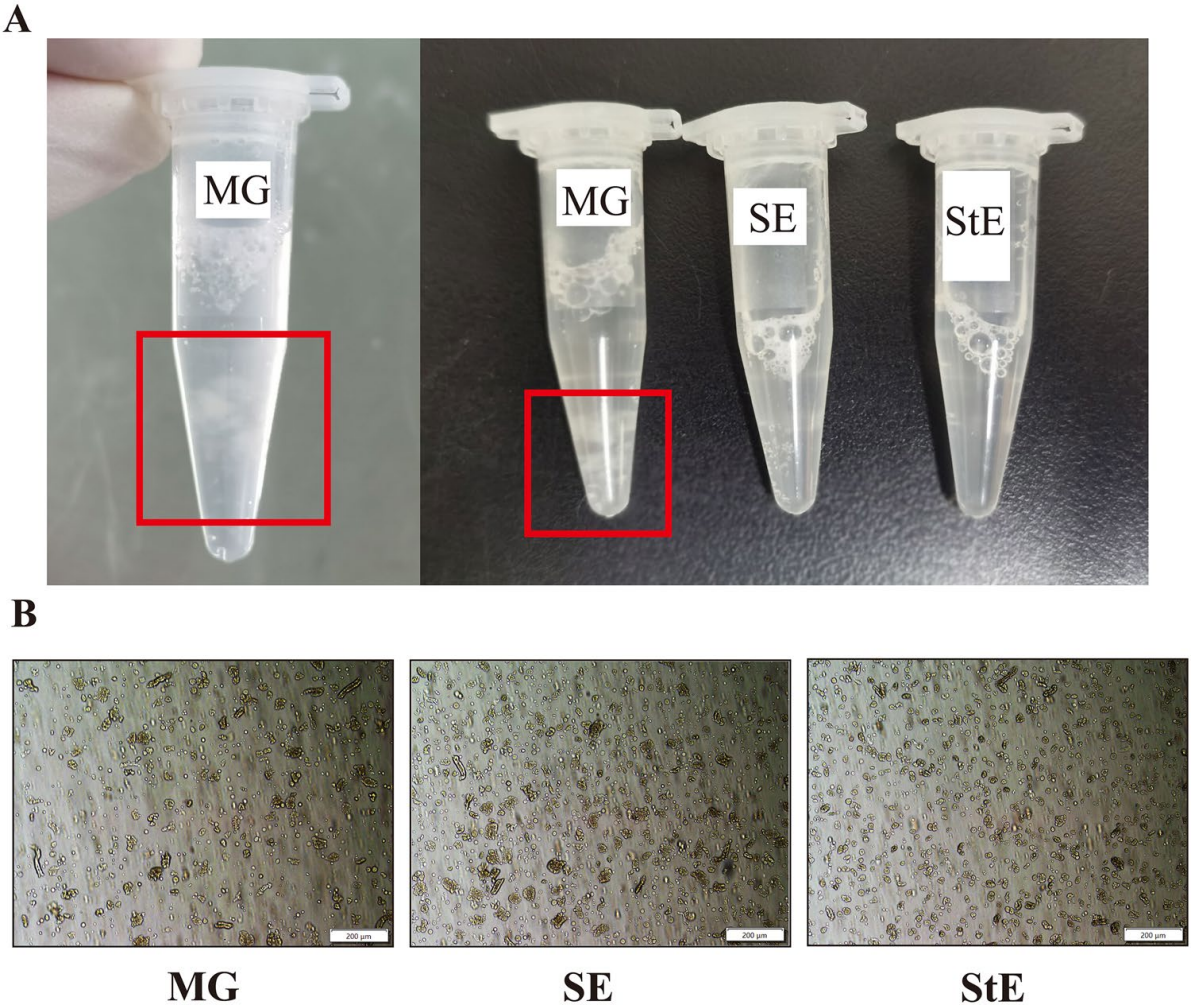
Morphological differences of cell suspensions in the different groups. (**A**) White flocculent suspended matter was observed in the supernatant of the MG group, while the upper liquid was clarified in the SE group and StE group. (**B**) The morphology of single-cell suspensions in the MG group, SE group and StE group was observed and photographed under a microscope. The magnification is × 100.

During the preparation of the single-cell suspension, the cells were observed in EP tubes after centrifugation. White flocculent suspended matter was visible in the supernatant in the MG group, while the upper liquid in the SE and StE groups was clarified (Fig. 1A). The suspended matter may have been the noncellular collagen component filtered through the strainer because of mechanical grinding that then reclustered after centrifugation.

The morphology of the single-cell suspensions of the MG, SE and StE groups was observed under an inverted microscope. Under 100× magnification, the cell dispersion of the MG group was fine, but there were many tissue fragments that varied in size and shape and there were more cell fragments smaller than cells. Some cells had poor integrity, and cell boundaries were not clear. The SE group had fewer cell fragments, but there were still some tissue fragments larger than cells. The StE group had fewer tissue fragments and fewer cell fragments than the other two groups. The cells of this group were well dispersed, and most of them had regular morphology and clear boundaries (Fig. 1B).

### Number of cells obtained per 1 g of kidney tissue and cell viability

One gram of kidney tissue was weighed for each group, single-cell suspensions were prepared by the above three methods, and the numbers of cells obtained were compared. The average number of cells harvested from each sample was 1.47 × 10^7^/g for the MG group, 2.36 × 10^7^/g for the SE group and 1.92 × 10^7^/g for the StE group. The numbers of harvested cells in the SE group and StE group were significantly greater than that in the MG group. There was no significant difference between the SE group and StE group. The cell viability was detected by flow cytometry using Zombie NIR Dye. The average cell viability was 73.19% for the MG group, 83.09% for the SE group, and 82.3% for the StE group. The values for the latter two groups were significantly higher than that for the MG group (Table 2).

**Table 2:** Number of cells obtained per 1 g of kidney tissue and cell viability.

Group	n	Total count (× 10^7^/g) (mean (SD))	Cell viability (%) (mean (SD))	Operation time (min) (mean (SD))
MG	5	1.47 (0.23)	73.19 (6. 12)	34.00 (2.12)
SE	5	2.36 (0.48)^*^	83.09 (3. 41)^*^	67.80 (0.84)^*^
StE	5	1.92 (0.14)^*^	82.3 (3. 14)^*^	69.00 (1.58)^*^

*p < 0.05 versus MG.

### Time consumption

The average operation time of MG was 34.00 min. Those of SE and StE were 67.80 min and 69.00 min, respectively, which was significantly more than that of MG. There was no significant difference between the SE and StE groups (Table 2).

### Percentage of singlets in each group

**Figure 2:**
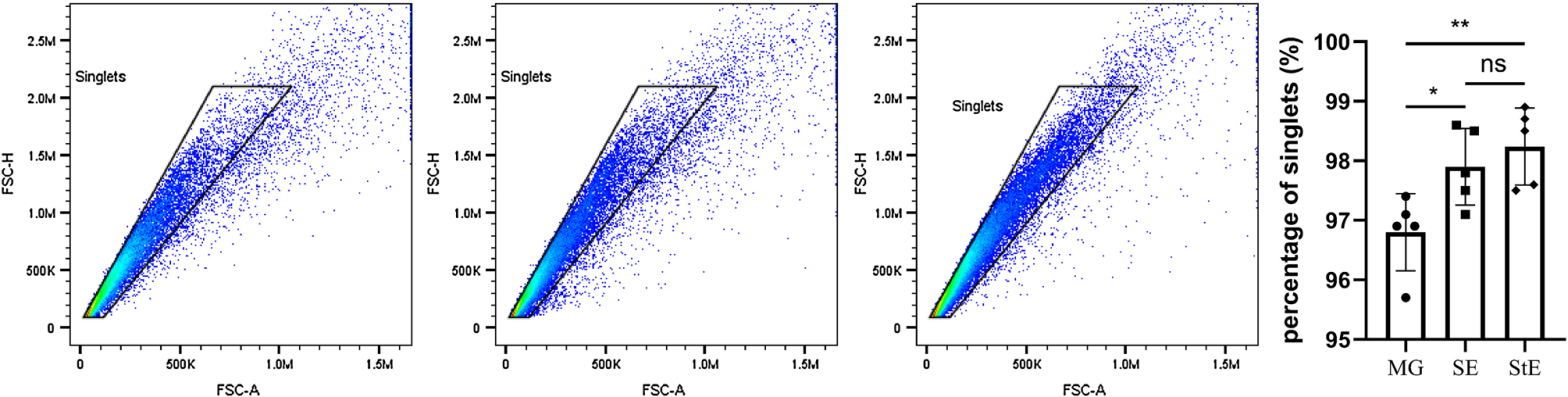
Percentage of singlets in each group. The singlet percentages of the SE and StE groups were significantly higher than that of the MG group, n = 5, *p < 0.05, **p < 0.01.

During preparation of single-cell suspensions, adhesions of two or more cells may form. These are collectively referred to as doublets and will affect the results of follow-up experiments such as single-cell sequencing and flow cytometry. A higher percentage of singlets is beneficial to the accuracy of the experimental results^11^. The results of flow cytometry analysis showed that the proportions of singlets in the SE and StE groups were significantly higher than that in the MG group, but there was no difference between the SE and StE groups (Fig. 2).

### Main cell types detected by flow cytometry

**Figure 3:**
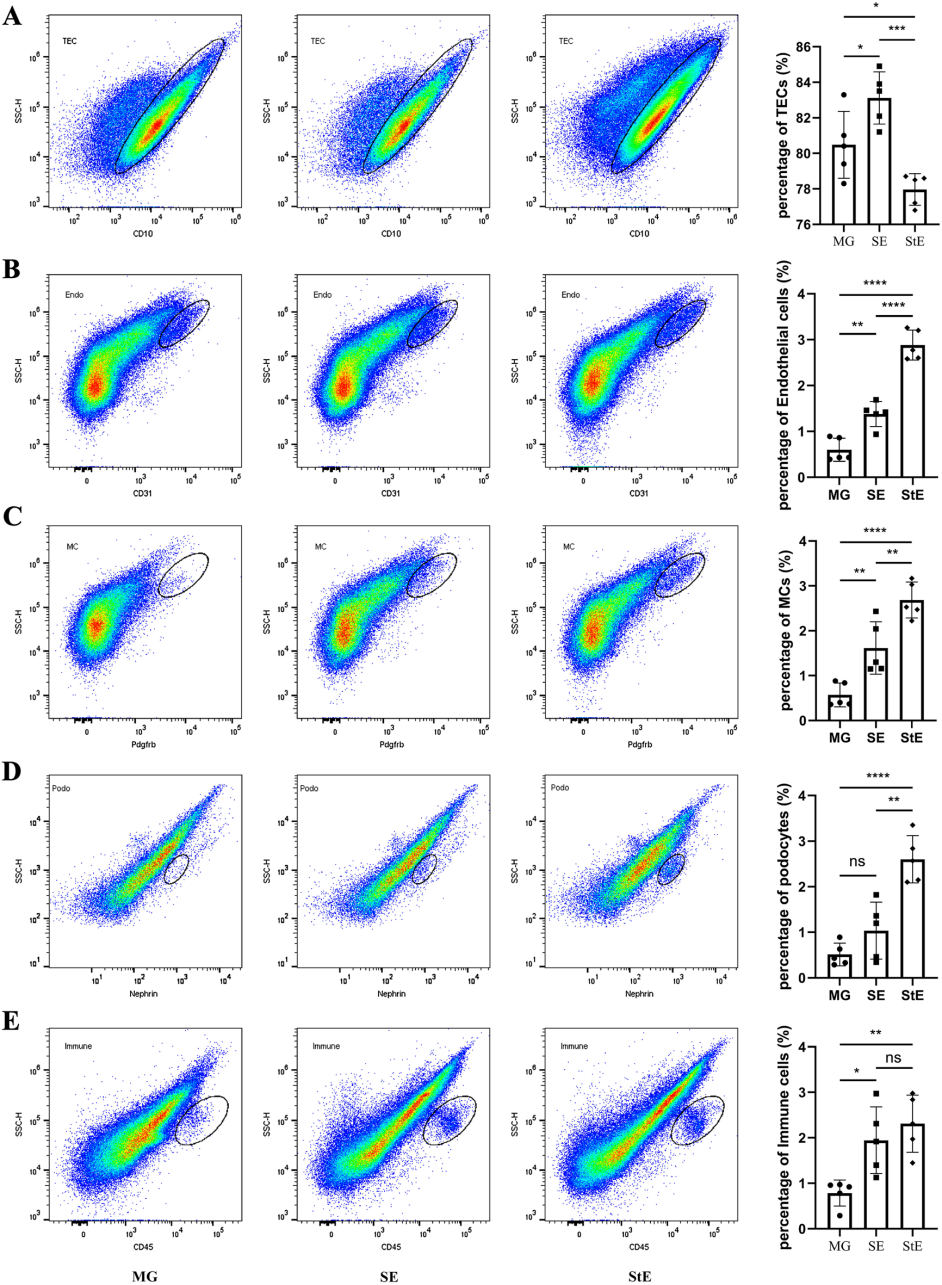
Proportions of different types of cells in the different groups. (**A**) The detection rate of renal tubular epithelial cells was highest in the SE group, followed by the MG group and then the StE group. (**B**,**C**) The detection rate of endothelial cells or mesangial cells was the highest in the StE group, followed by the SE and MG groups. (**D**) The detection rate of podocytes was significantly higher in the StE group than in the MG and SE groups, with no difference between the latter two. (**E**) The detection rates of immune cells in the SE and StE groups were significantly higher than that in the MG group, with no difference between the first two groups, n = 5, *p < 0.05, **p < 0.01, ***p < 0.001, ****p < 0.0001. TECs, renal tubular epithelial cells; MCs, mesangial cells; Podo, podocytes; Endo, endothelial cells.

The main cell types in renal tissue were detected by flow cytometry and the gating strategy could be seen in Fig. S1. The detection rate of renal tubular epithelial cells was the highest in the SE group, followed by the MG group and then the StE group. There were significant differences among all groups. The detection rates of endothelial cells and mesangial cells were the highest in the StE group, followed by the SE and MG groups, and there were significant differences among all groups. Podocytes were significantly more abundant in the StE group than in the MG and SE groups, and there was no difference between the latter two. Immune cells were significantly more abundant in the SE and StE groups than in the MG group, and there was no difference between the first two groups (Fig. 3).

### Main cell types detected by qRT-PCR

**Figure 4:**
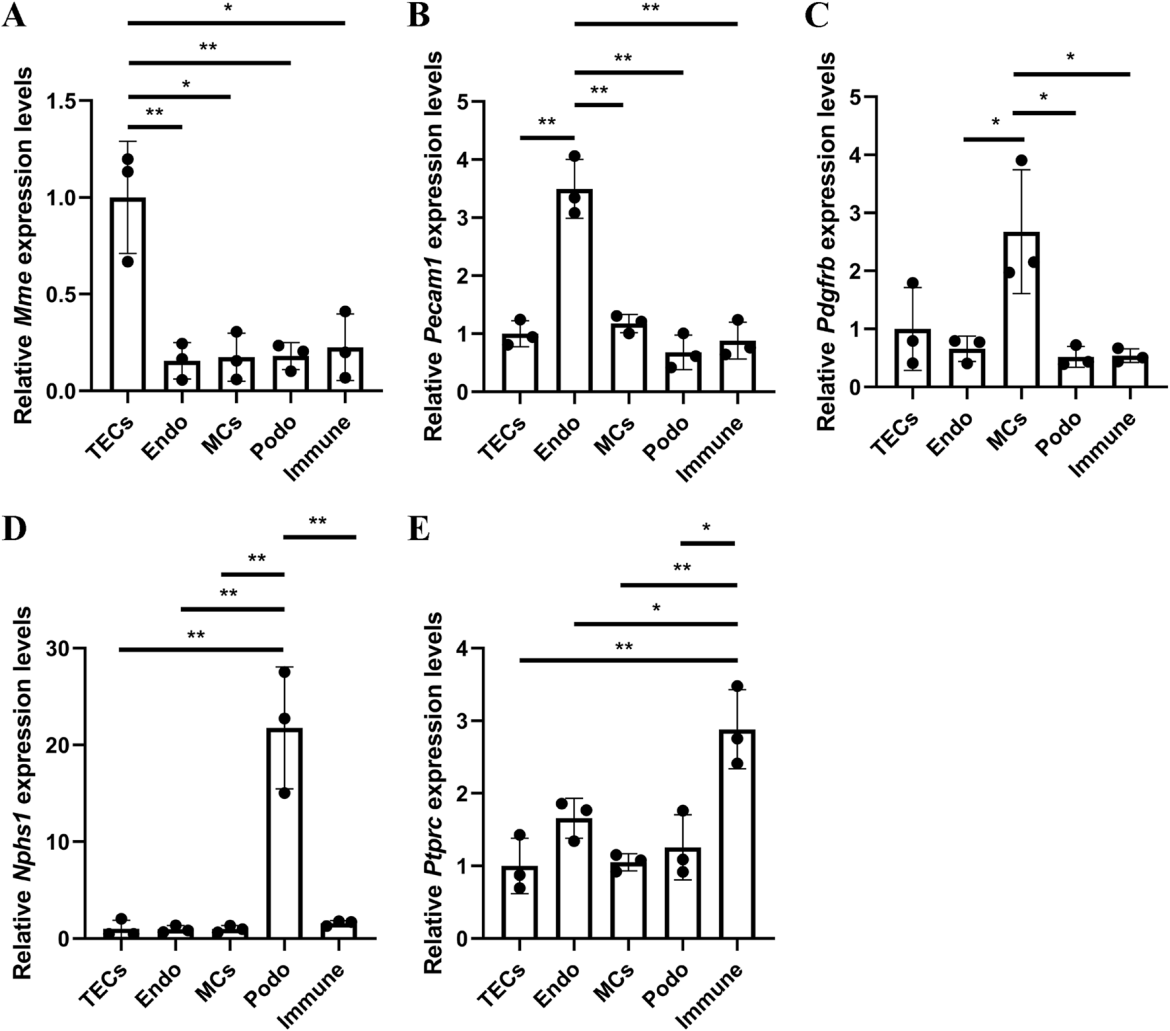
The expressions of *Mme*, *Pecam1*, *Pdgfrb*, *Nphs1* and *Ptprc* in each type of sorted cells detected by qRT-PCR. (**A**) The expression of *Mme* in renal tubular epithelial cells was higher than that of the others. (**B**) The expression of *Pecam1* in endothelial cells was higher than that of the others. (**C**) The expression of *Pdgfrb* in mesangial cells was higher than that of the others. (**D**) The expression of *Nphs1* in podocytes was higher than that of the others. (**E**) The expression of *Ptprc* in immune cells was higher than that of the others. n = 3, *p < 0.05, **p < 0.01. TECs, renal tubular epithelial cells; MCs, mesangial cells; Podo, podocytes; Endo, endothelial cells.

*Mme*, *Pecam1*, *Pdgfrb*, *Nphs1*, *Ptprc* are the genes encoding the CD10, CD31, Pdgfrb, Nephrin, CD45, which are the markers of renal tubular epithelial cells, endothelial cells, mesangial cells, podocytes and immune cells in turn. The qRT-PCR results showed that the expression of the marker was significantly higher in this type of cells than the others at RNA level, which proved that the sorted cell types were exact (Fig. 4).

## Discussion

The kidneys are highly complex organs, and nephrons are their basic constituent units. Nephrons include several cell types, including the epithelial cells that make up the renal tubules and the endothelial cells, mesangial cells and podocytes that make up the glomeruli^6^. There are also a certain number of resident immune cells in the kidneys that maintain the homeostasis of the organ and play a role in organ regional immunity^12^. With the development and maturation of single-cell sequencing technology^13^, it has become necessary to develop a convenient and efficient method for the preparation of whole-kidney single-cell suspensions, which will form the basis for understanding the structural function of the kidneys at the single-cell level.

Renal tubules occupy 80% to 90% of the volume of the kidneys, and the proportion of renal tubular epithelial cells can exceed 80%^14^. The basement membrane of renal tubules is relatively thin and is easier for digestive enzymes to dissolve than other structures. This is the reason why tubule cells are relatively easy to obtain when preparing single-cell suspensions. However, because of the large number of renal tubule cells and the ease of their digestion, tubule cells can easily mask the presence of other cells, especially rare cell types and their subtypes, in prepared single-cell suspensions of kidney tissue^8^. The glomerular basement membrane (GBM) is an important part of the glomerular capillary wall, whose main components include laminin, type IV collagen, nidogen and heparin sulfate proteoglycan. Type IV collagen is the main component of all basement membranes and is considered to provide tensile and compressive strength^15^. Mesangial cells and the mesangial matrix provide structural support for the glomerular capillary loops, and the main mesangial matrix component is also type IV collagen^16^. Therefore, according to the characteristics of this structural component, previous studies have also used type IV collagenase to digest the basement membrane of the glomeruli to obtain single-cell suspensions of glomeruli^17^. However, the preliminary experimental results of this study showed that type IV collagenase alone took nearly 2 h to destroy the glomerular structure, with considerable loss of the easily digestible renal tubular epithelial cells due to overdigestion. Previous studies have used MTDK2 reagent to digest kidney tissue from rodents^6^. This reagent is a compound enzyme whose creation requires mixing of various components before use. MTDK2 reagent can fully dissociate the kidney matrix, has a gentle effect and can largely avoid cell damage; thus, it is widely used in the preparation of single-cell suspensions of kidney tissue from rats and mice^6,18^. However, studies have also shown that the numbers of podocytes, mesangial cells, and endothelial cells in kidney tissue dissociated with this reagent are reduced^19^. To compensate for this defect, we added type IV collagenase to the MTDK2 reagent when the initial dissociation of the renal tubules occurred around the glomeruli. Treatment of type IV collagen, which is enriched in the GBM and glomerular mesangial region, made it easier for glomeruli to split and facilitated the acquisition of single glomerular cells. The diameter of human glomeruli is between 100 and 250 μm^20^. In our pre-experiment, the average glomerular diameter of wild-type Wistar rats was approximately 100 μm (Fig. S2). The diameter of renal tubules after digestion is 40 μm or less^21^. Therefore, the use of a 40 μm cell strainer can essentially ensure that the cell suspension is composed of singlets. According to the results of this study, the proportions of singlets in the SE and StE groups were higher than that in the MG group, suggesting that a purer singlet suspension was obtained by enzymatic digestion than by MG.

The MG method tested in this study is also applied for the preparation of kidney single-cell suspensions and has the advantages of simple operation and low cost. However, the proportion of doublets in the single-cell suspension prepared by MG was relatively high, the acquisition rates of various types of glomerular and immune cells were low, the cell damage was severe, and there were many tissue fragments in the suspension. The above results may have been caused by the difficulty of thorough grinding, and collagen components may have passed through the strainer due to extrusion and formed flocculants after centrifugation.

To verify the numbers of various types of cells in glomeruli and tubules obtained by the different preparation methods, we examined the classical markers of these cells, including the endothelial cell marker CD31^22^, the podocyte marker nephrin^23^, the mesangial cell marker PDGFR beta and the renal tubular epithelial cell marker CD10^24^. The results showed that StE significantly increased the acquisition rates of endothelial cells, mesangial cells and podocytes in glomeruli. There are many kinds of immune cells in the kidneys, which can be divided into circulating immune cells and resident immune cells, including monocytes/macrophages, T lymphocytes, B lymphocytes and NK cells^25^. CD45, also known as leukocyte common antigen (LCA), is highly expressed in immune cells and can be used as a marker of immune cells^26^. The results also showed that StE had the highest acquisition rate of immune cells and suggest that StE is a suitable method for the preparation of single-cell suspensions for research on resident immune cells in renal tissue.

**Figure 5:**
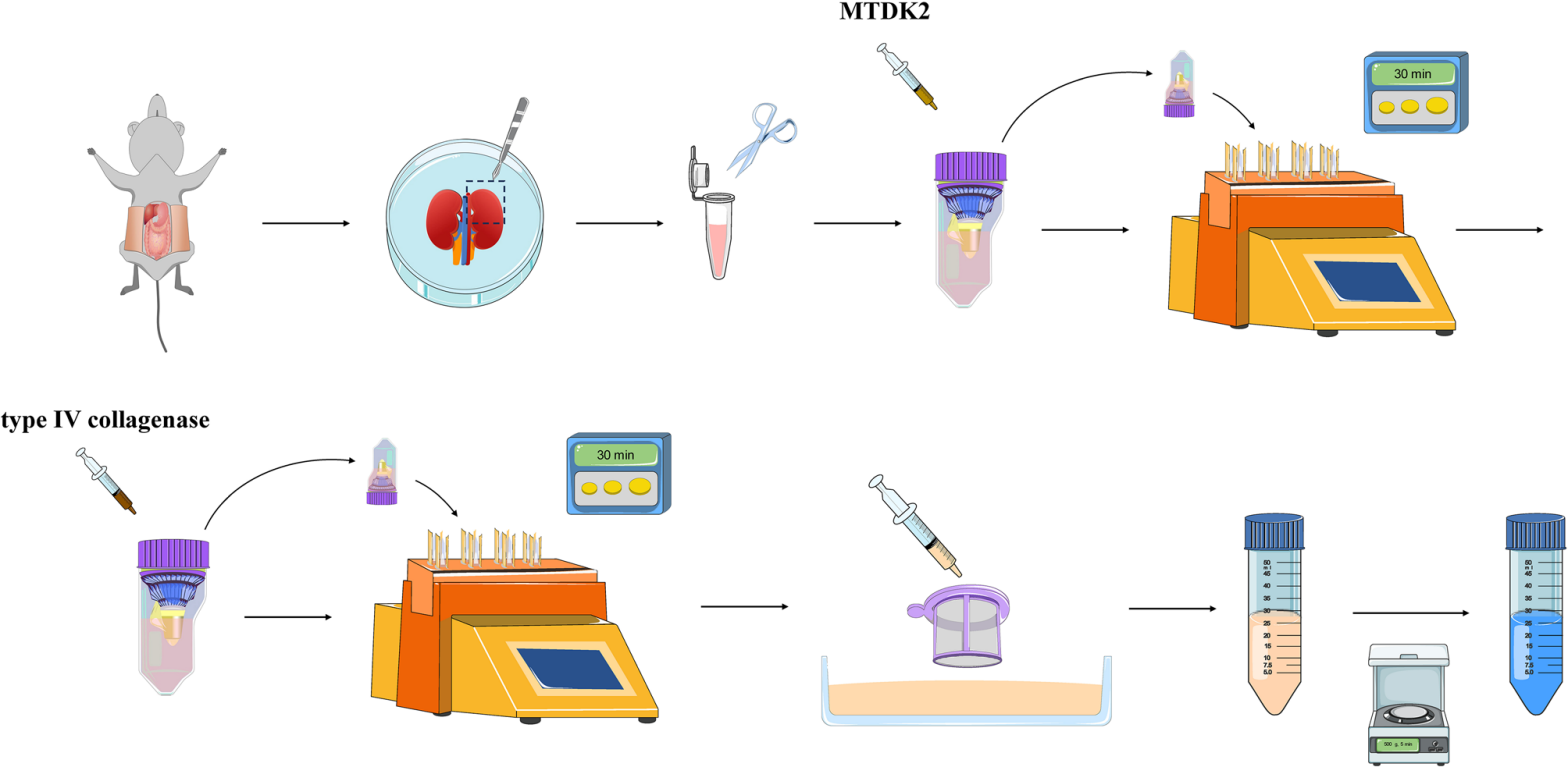
Schematic description of stepwise enzymatic digestion method.

In summary, based on previous experimental methods, we here designed a new preparation method for whole-kidney tissue single-cell suspensions (Fig. 5) that can not only ensure the acquisition of renal tubular epithelial cells with high viability but also enable harvest of more intrinsic glomerular cells and immune cells in kidney tissue. In other words, the StE method balanced the bias of cell acquisition caused by insufficient digestion and the decrease in cell viability caused by excessive digestion and improved the acquisition rates of intrinsic cells of glomeruli and resident immune cells under the condition of high cell viability. In addition, this method is low-cost and only moderately time-consuming, and it relies on common laboratory equipment, reagents and consumables. This method is feasible, highly efficient, and has considerable potential for popularization.

### Supplementary Information

Supplementary Figures.

## Data Availability

All data generated or analysed during this study are included in this published article [and its supplementary information files].

## References

[1] Yamada K (2001). Clusterin is up-regulated in glomerular mesangial cells in complement-mediated injury. Kidney Int..

[2] Amon L (2022). Guidelines for DC preparation and flow cytometry analysis of mouse lymphohematopoietic tissues. Eur. J. Immunol..

[3] Yaigoub H (2022). Isolation of viable single cells with high yield and purity using a small amount of human kidney tissue biopsy. Front. Cell Dev. Biol..

[4] Teal E, Steele NG, Chakrabarti J, Holokai L, Zavros Y (2018). Mouse-and human-derived primary gastric epithelial monolayer culture for the study of regeneration. J. Vis. Exp..

[5] Guyton AC (1991). Blood pressure control–special role of the kidneys and body fluids. Science.

[6] Park J (2018). Single-cell transcriptomics of the mouse kidney reveals potential cellular targets of kidney disease. Science.

[7] Balzer MS, Rohacs T, Susztak K (2022). How many cell types are in the kidney and what do they do?. Ann. Rev. Physiol.

[8] Young MD (2018). Single-cell transcriptomes from human kidneys reveal the cellular identity of renal tumors. Science.

[9] Baldelomar EJ (2016). Phenotyping by magnetic resonance imaging nondestructively measures glomerular number and volume distribution in mice with and without nephron reduction. Kidney Int..

[10] Onoda N (2022). Spatial and single-cell transcriptome analysis reveals changes in gene expression in response to drug perturbation in rat kidney. DNA Res..

[11] McGinnis CS, Murrow LM, Gartner ZJ (2019). DoubletFinder: Doublet detection in single-Cell RNA sequencing data using artificial nearest neighbors. Cell Syst..

[12] Kurts C, Panzer U, Anders HJ, Rees AJ (2013). The immune system and kidney disease: Basic concepts and clinical implications. Nat. Rev. Immunol..

[13] Macosko EZ (2015). Highly parallel genome-wide expression profiling of individual cells using nanoliter droplets. Cell.

[14] Liao J (2020). Single-cell RNA sequencing of human kidney. Sci. Data.

[15] Naylor RW, Morais M, Lennon R (2021). Complexities of the glomerular basement membrane. Nat. Rev. Nephrol..

[16] Schlondorff D, Banas B (2009). The mesangial cell revisited. J. Am. Soc. Nephrol..

[17] Zambrano S (2022). Molecular insights into the early stage of glomerular injury in IgA nephropathy using single-cell RNA sequencing. Kidney Int..

[18] Marelli-Berg FM, Peek E, Lidington EA, Stauss HJ, Lechler RI (2000). Isolation of endothelial cells from murine tissue. J. Immunol. Methods.

[19] Denisenko E (2020). Systematic assessment of tissue dissociation and storage biases in single-cell and single-nucleus RNA-seq workflows. Genome Biol..

[20] Samuel T, Hoy WE, Douglas-Denton R, Hughson MD, Bertram JF (2007). Applicability of the glomerular size distribution coefficient in assessing human glomerular volume: The Weibel and Gomez method revisited. J. Anat..

[21] Korin B, Chung JJ, Avraham S, Shaw AS (2021). Preparation of single-cell suspensions of mouse glomeruli for high-throughput analysis. Nat. Protoc..

[22] Akis N, Madaio MP (2004). Isolation, culture, and characterization of endothelial cells from mouse glomeruli. Kidney Int..

[23] Saleem MA (2002). A conditionally immortalized human podocyte cell line demonstrating nephrin and podocin expression. J. Am. Soc. Nephrol..

[24] Kuppe C (2021). Decoding myofibroblast origins in human kidney fibrosis. Nature.

[25] Stewart BJ (2019). Spatiotemporal immune zonation of the human kidney. Science.

[26] Alexander DR (2000). The CD45 tyrosine phosphatase: A positive and negative regulator of immune cell function. Semin. Immunol..

