# Supplementary material for: Comparison of preparation methods of rat kidney single-cell suspensions
Source: Sci Rep. 2024 Feb 2;14:2785. doi: 10.1038/s41598-024-53270-2 (PMC10837120; doi:10.1038/s41598-024-53270-2)
Supplement: Supplementary file 1 — Supplementary Figures. [file 41598_2024_53270_MOESM1_ESM.docx]

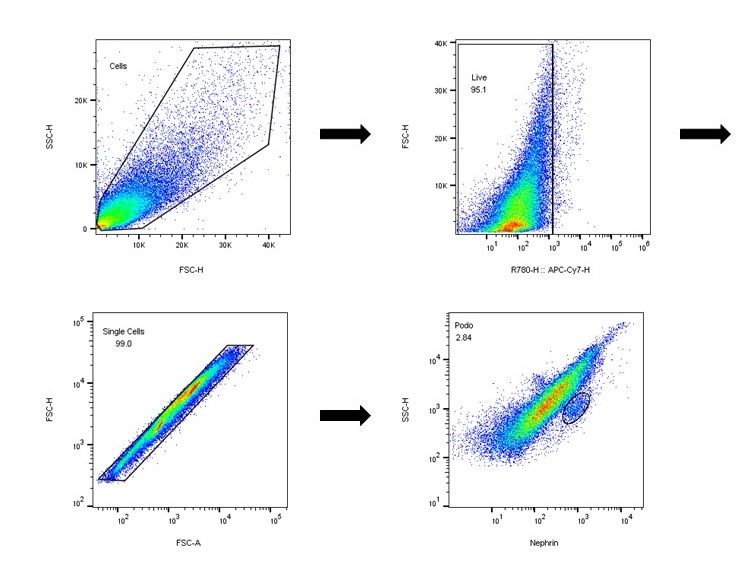


**Figure S1.** The gating strategy of a particular group of cells. Take the podocytes as example. 1. gating the cells without debris; 2. gating the live cells; 3. gating the singlets; 4. gating the interested cells


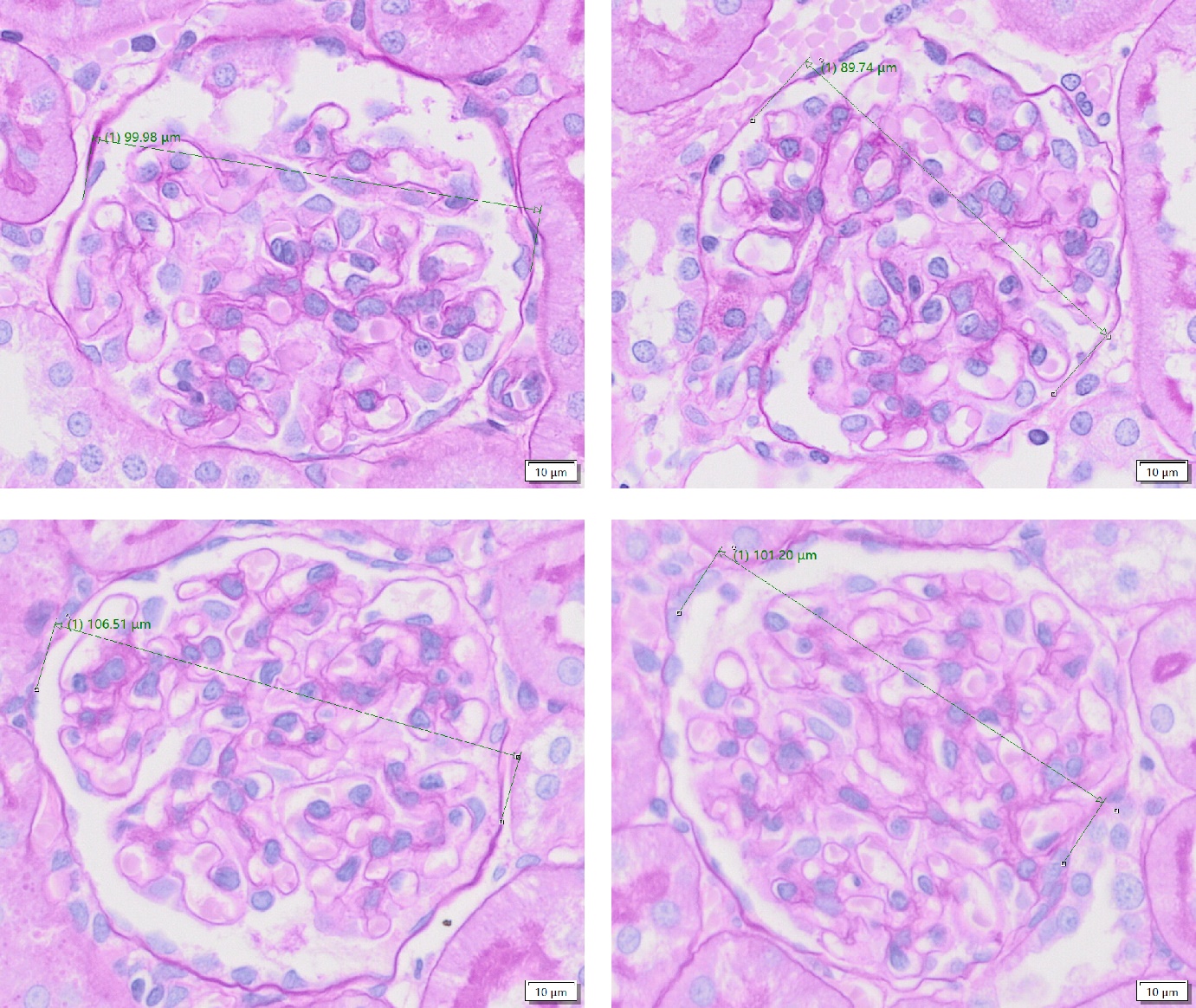


**Figure S2.** Glomerular diameter measurement. For example, these four glomerular diameters were 99.98 μm, 89.74 μm, 106.51 μm, 101.20 μm. The average length was approximately 100 μm.
